# The miR-125a/HK2 axis regulates cancer cell energy metabolism reprogramming in hepatocellular carcinoma

**DOI:** 10.1038/s41598-017-03407-3

**Published:** 2017-06-08

**Authors:** Fangfang Jin, Yanbo Wang, Yanan Zhu, Shan Li, Ying Liu, Cheng Chen, Xiaohua Wang, Ke Zen, Limin Li

**Affiliations:** 1 State Key Laboratory of Pharmaceutical Biotechnology, Nanjing University Advanced Institute of Life Sciences, Jiangsu Engineering Research Center for MicroRNA Biology and Biotechnology, Nanjing University, Nanjing, Jiangsu 210093 China; 2 Department of Chemotherapy, Jiangsu Cancer Hospital and Research Institute, Nanjing, Jiangsu 210009 China; 3 Department of Radiotherapy, Nanjing Medical University Affiliated Cancer Hospital, Cancer Institute of Jiangsu Province, Nanjing, Jiangsu 210009 China

## Abstract

The Warburg effect is a metabolic hallmark of cancer. Tumor cells rapidly adjust their energy source to glycolysis in order to efficiently proliferate in a hypoxic environment, but the mechanism underlying this switch remains incompletely understood. Here, we show that hypoxia potently induces the down-regulation of miR-125a expression in hepatocellular carcinoma (HCC) cells and tumors. Furthermore, we demonstrate that miR-125a could decrease the production of lactate, the uptake of glucose, and the levels of ATP and reactive oxygen species (ROS) in HCC cells. We investigated the molecular mechanism through which miR-125a inhibits HCC glycolysis and identified hexokinase II (HK2) as a direct target gene of miR-125a. Finally, we revealed that the miR-125a/HK2 axis is functionally important for regulating glycolysis of HCC cell and progression of cancer *in vitro* and *in vivo*. In summary, our findings demonstrate for the first time that hypoxia-down-regulated miR-125a regulated HCC glycolysis and carcinogenesis by targeting hexokinase HK2, a key glycolytic enzyme for the Warburg effect, and add a new dimension to hypoxia-mediated regulation of cancer metabolism.

## Introduction

Hepatocellular carcinoma (HCC) is the third most common cause of cancer-related death, and the incidence of HCC is expected to increase worldwide^1^. As an inherent feature of solid tumors, including HCC, hypoxia or inadequate oxygen supply contributes to cancer metabolism reprogramming. To sustain continuous growth and proliferation, tumor cells also modify their metabolism to adapt to challenging hypoxic environments^2^. The Warburg effect, where by cellular energy production is driven by glycolysis even in the face of oxygen levels that are sufficient to support oxidative phosphorylation, is a core metabolic hallmark of cancer^3^. In addition to generating ATP, this metabolic reprogramming provides glycolytic intermediates needed to address the biosynthetic needs of fast-growing tumors^4–7^.

Tumor prefer low oxygen levels and utilize glycolysis to generate ATP^2, 3^. Thus targeting metabolism of HCC, especially in hypoxic environments, is likely to be of great value for HCC treatment. Hexokinases catalyze the first and irreversible step of glucose metabolism (ATP-dependent phosphorylation of glucose to yield glucose-6-phosphate)^8^. Hexokinase 2 (HK2) is the major isozyme that is abundantly expressed in a variety of cancers and contributes to aerobic glycolysis, and thus it is reported as a pivotal player in the Warburg effect and is proposed as a metabolic target for cancer therapeutic development^9, 10^. However, little is known about the regulatory mechanisms of HK2 in HCC energy metabolism so far.

In the past decade, a class of small non-coding RNAs known as microRNAs (miRNAs) has emerged as a major regulator of the initiation and progression of human cancers^11, 12^. miRNAs regulate gene expression at the post-transcriptional level by binding to the 3′-untranslated regions (UTRs) of target mRNAs to either block mRNA translation or trigger mRNA degradation. Dysregulated and dysfunctional miRNAs play a causal role in cancer etiology because miRNAs can regulate targeted oncogenes and tumor suppressors^11, 13^. Recently, a series of miRNAs has been found to be involved in the regulation of tumor energy metabolism^14–16^, however, our understanding of the role of miRNAs in the glycolytic switch in HCC remains limited. Thus far, only some miRNAs, including miR-129^17^, miR-199a^18, 19^, miR-338^20^, and miR-23a^21^, have been confirmed to modulate the HCC Warburg effect. Little is known about how the Warburg effect is regulated by miRNAs and how it contributes to hepatocarcinogenesis.

In this study, we found for the first time that the down-regulation of miR-125a is crucial for the glycolysis-promoting effect of hypoxia in human HCC cells and tumors. Down-regulation of miR-125a promotes the Warburg effect of HCC by directly inhibiting the expression of hexokinase HK2, a critical glycolytic enzyme in the Warburg effect^18, 22, 23^. We focused on the functional significance and regulatory mechanisms of the miR-125a/HK2 axis in the regulation of the Warburg effect and tumor growth *in vitro* and *in vivo*. These results provide new insights into the role of the miR-125a/HK2 axis in the glycolytic switch of HCC and add a new dimension to hypoxia-mediated regulation of cancer metabolism.

## Results

### Hypoxia induced miR-125a down-regulation in HCC cells and tumors

**Figure 1:**
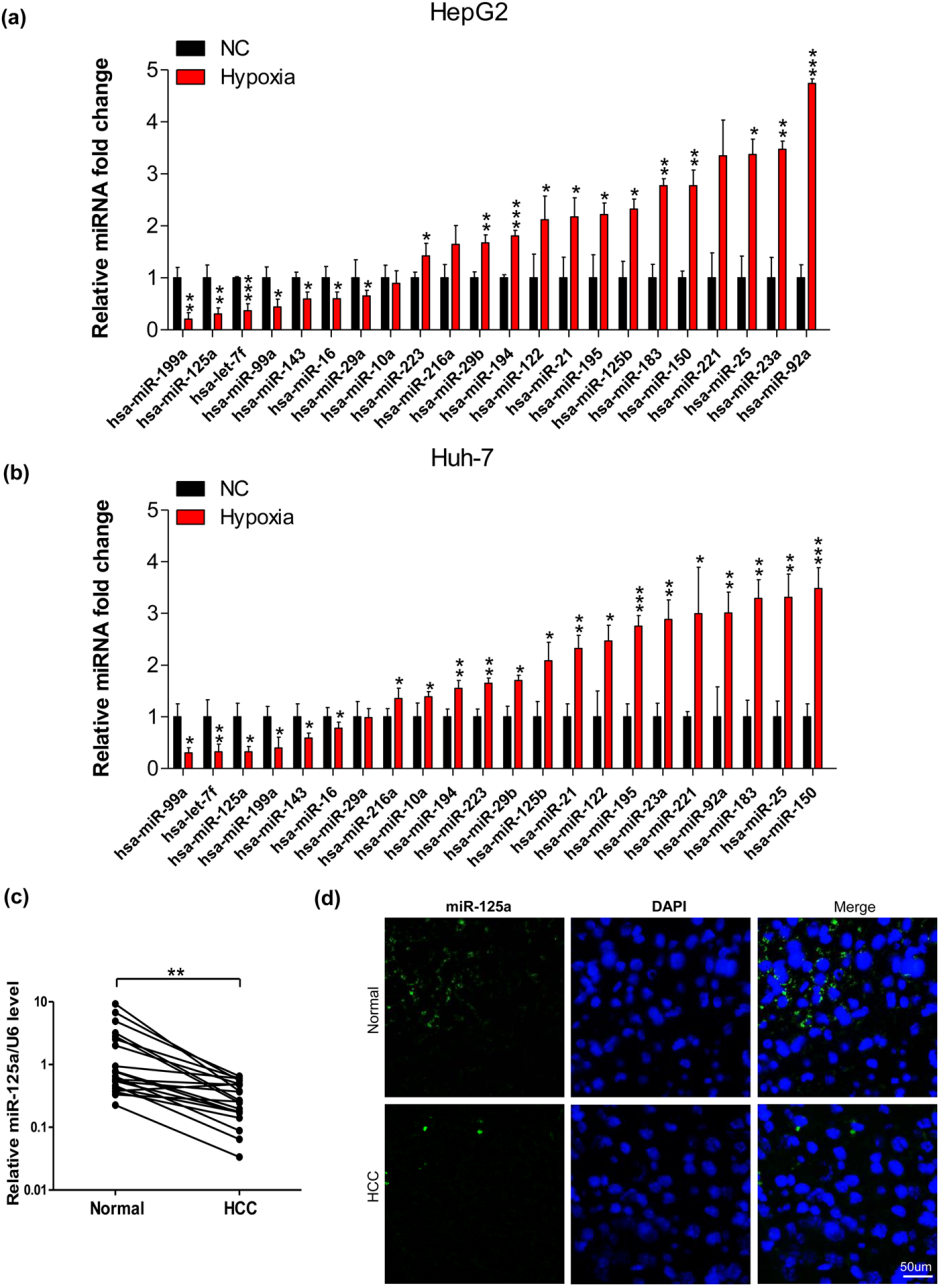
Hypoxia down-regulates miR-125a in HCC cells and tumors. **(a**,**b**) Relative levels of 22 miRNAs in HepG2 and Huh-7 cells after treatment with hypoxia for 48 h were measured using RT-qPCR. (**c**) Relative levels of miR-125a (expressed as the miRNA/U6 ratio) in paired tumor tissues and normal tissues were determined using RT-qPCR (n = 21). (**d**) Localization of miR-125a in paired tumor tissues (HCC) and normal tissues (Normal) from HCC patients were analyzed by *in situ* hybridization. All data are shown as the means ± S.E. obtained from three separate experiments.

To explore hypoxia-regulated cancer metabolism, we used RT-qPCR to compare the expression of 22 liver cancer-related miRNAs in HepG2 and Huh-7, with and without hypoxia treatment^24–26^. Immunoblot analysis confirmed that HIF-1α was rapidly induced by hypoxia in HCC cells (Supplementary Fig. 1a and b). Interestingly, hypoxia led to a common down-regulation of 6 miRNAs (miR-199a, miR-125a, let-7f, miR-99a, miR-143 and miR-16). miR-199a and miR-125a, among all the tested miRNAs, had the greatest reduction under hypoxia treatment (Fig. 1a and b), consistent with a previous observation in SMMC-7721 and Hep3B cells^18^. Moreover, we measured the levels of miR-125a in 21 pairs of HCC samples and adjacent noncancerous tissues. RT-qPCR and i*n situ* hybridization results clearly showing that miR-125a was consistently down-regulated in HCC tumor tissues compared to the corresponding background tissues (Fig. 1c and d). Taken together, these results imply that down-regulation of miR-125a is important to hypoxia-induced cellular responses.

### Down-regulation of miR-125a promotes glycolysis in HCC cells under hypoxia

**Figure 2:**
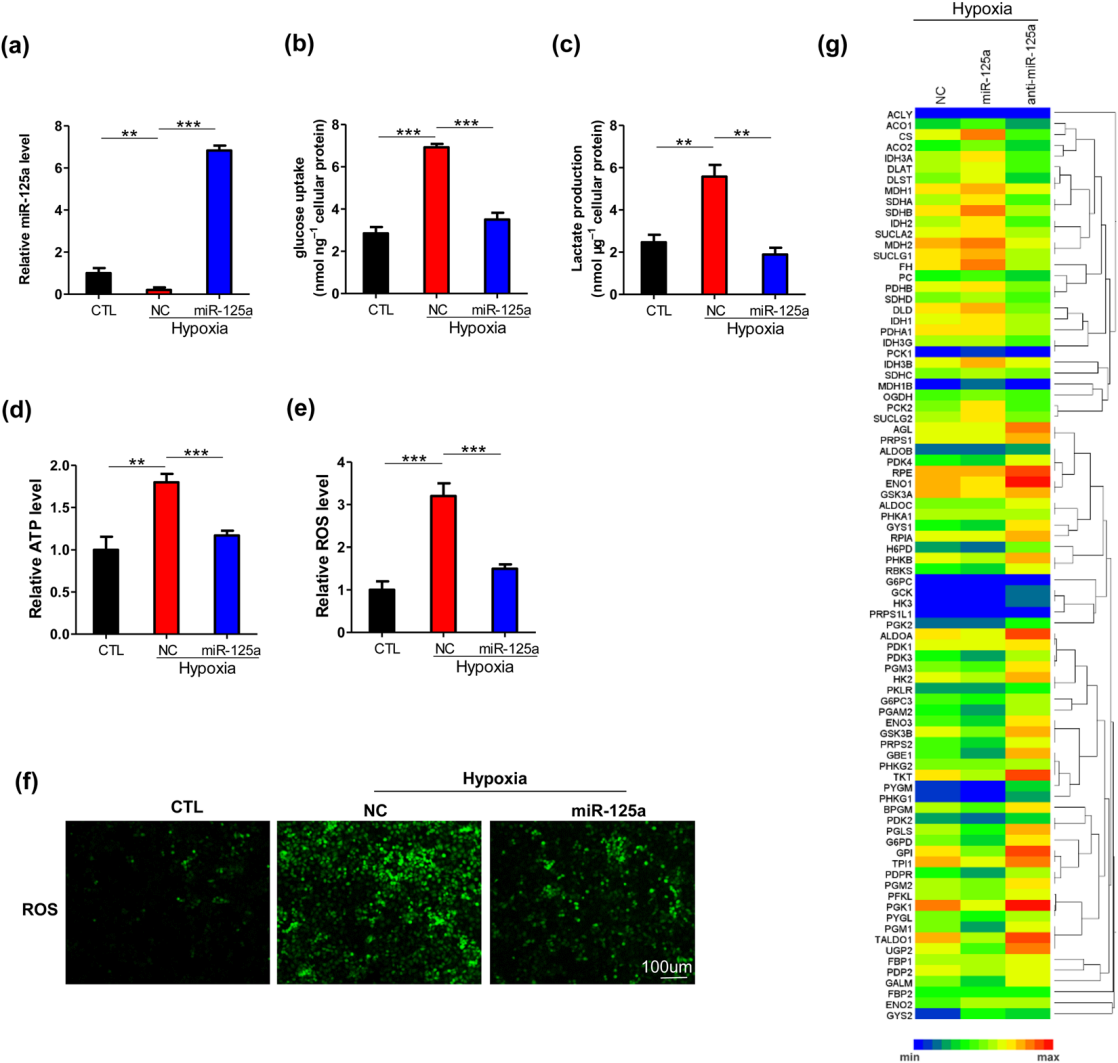
Hypoxia promotes glucose metabolism in HCC cell via down-regulating miR-125a. Restoration of miR-125a expression rescued the glycolysis-promoting effect of hypoxia in HepG2 cells. Relative levels of miR-125a (**a**), the uptake of glucose (**b**), the production of lactate (**c**), cellular levels of ATP (**d**) and ROS (**e**) were evaluated. (**f**) ROS was observed by fluorescence micrography in HepG2 cells under normoxia or hypoxia transfected with/without miR-NC, miR-125a mimics. (**g**) A total of 84 genes were quantified in HepG2 cells transfected with miR-125a mimics and inhibitors under hypoxia using a human glucose metabolism PCR array. All data are shown as the means ± S.E. obtained from three separate experiments.

Then, we examined whether down-regulation of miR-125a is involved in the metabolic response to hypoxia in HCC cells. Measurement of metabolic parameters revealed that the uptake of glucose (Fig. 2b) and the production of lactate (Fig. 2c) were increased significantly in HepG2 cells under a hypoxic state, indicating that hypoxia promotes glycolysis significantly. Because of glycolysis is less efficient than oxidative phosphorylation to production of ATP. To meet the demand of ATP, tumor cells have to develop alternatives pathways to increase ATP production^27^. In addition, ROS is a common toxic by-products of tumor cells to bypass cellular stress by changing the metabolism pathway under the condition of lack nutrients and oxygen. It has long been thought to aid tumor development in several ways^28^. Therefore, ATP and ROS level are important indicators for tumor survival, growth, and expansion. Therefore, we detected the levels of ATP and ROS under a hypoxic state. As shown in Fig. 2d,e and f, ATP (Fig. 2d) and ROS levels (Fig. 2e and f) were increased significantly in HepG2 cells under hypoxia. Moreover, restoration of miR-125a expression by transfecting miR-125a mimics reversed the impact of hypoxic stress on glucose metabolism (Fig. 2a–f).

To further validate the regulatory effect of miR-125a on glucose metabolism, we transfected HepG2 cells with miR-125a mimics and inhibitors under hypoxia, and then performed an RT^2^ Profiler PCR Array to detect the effect of miR-125a on the expression of genes involved in glucose metabolism, including glycolysis pathway, TCA cycle and so on. As shown in Fig. 2g, overexpression of miR-125a inhibits the expression of glycolysis-related genes, such as HK2, ALDOA and so on, compared with control cells (NC), whereas suppression of miR-125a promotes glycolysis pathway. Together, these results showed that down-regulation of miR-125a represents an important mechanism for the glycolysis-promoting effect of hypoxia in HCC cells.

### HK2 is a miR-125a direct target involved in the Warburg effect

**Figure 3:**
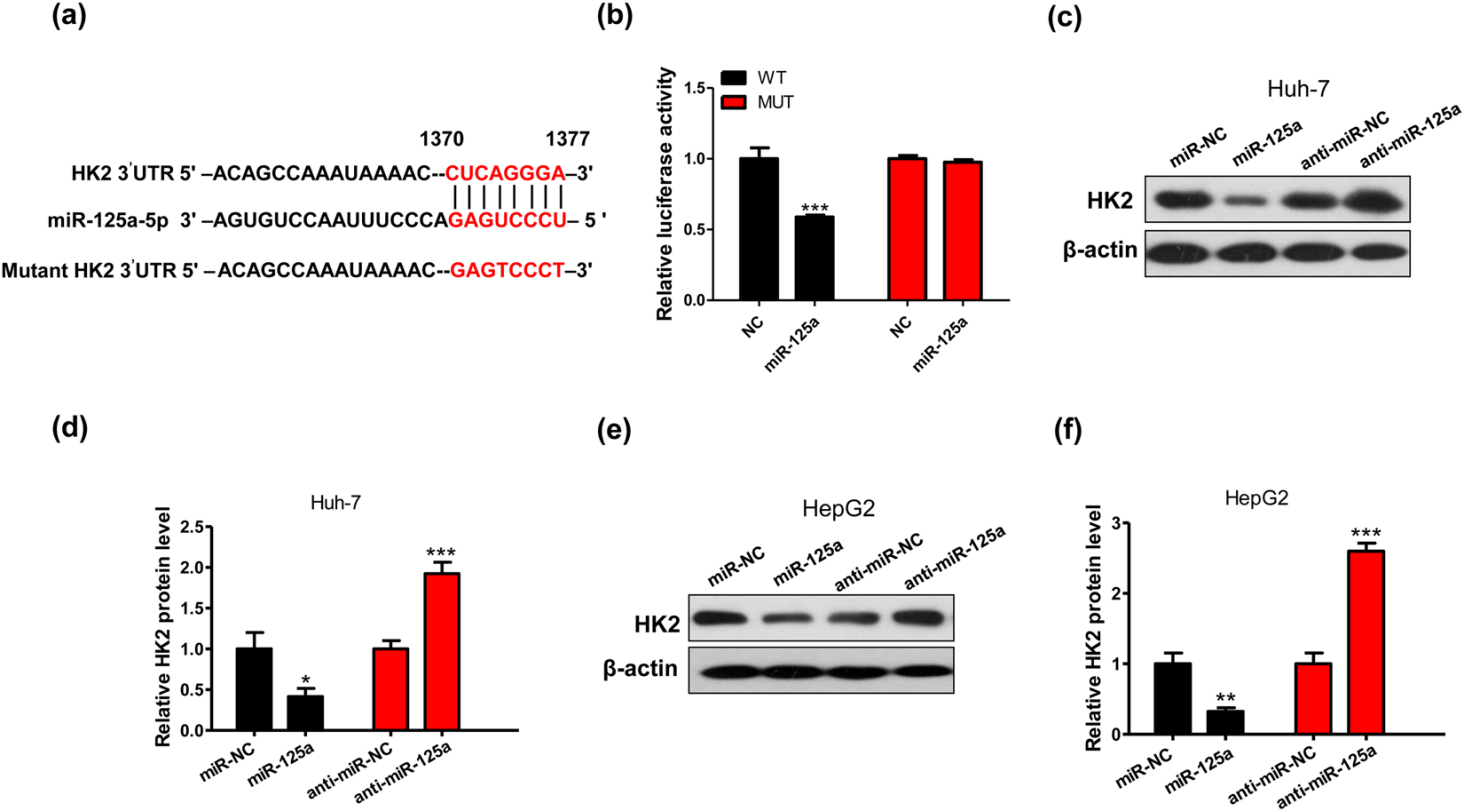
miR-125a directly targets HK2. (**a**) A schematic diagram of HK2 3′UTR as a putative target for miR-125a. The seed-recognizing sites and mutant sites are indicated in red. (**b**) Relative luciferase activity in 293T that were transfected with firefly luciferase reporters containing WT or mutant 3′ UTRs of HK2, miR-125a mimics or miR-NC. (**c**–**f**) Huh-7 and HepG2 cells were transfected with miR-NC, miR-125a mimics, anti-miR-NC or anti-miR-125a. The expression levels of HK2 were detected using immunoblotting.

To explore the molecular mechanisms underlying these phenomena, the candidate targets of miR-125a were predicted using a combination of three databases: TargetScan, miRanda and PicTar. The three servers consistently predicted HK2, a key glycolytic gene in the Warburg effect^14, 22, 23^ as the potential target of miR-125a. Thus, HK2 was chosen for further experimental validation. The predicted interaction between miR-125a and targeting sites within the 3′-UTR of HK2 are illustrated in Fig. 3a. To validate the binding of miR-125a to HK2 3′-UTR, a luciferase reporter assay was performed. The full-length 3′-UTR of HK2 containing the presumed binding sites for miR-125a was placed downstream of the firefly luciferase gene in a reporter plasmid. As anticipated, luciferase activity was remarkably reduced in cells co-transfected with the luciferase reporter plasmid and miR-125a mimics (Fig. 3b). Furthermore, we introduced point mutations into the corresponding complementary sites in the 3′-UTR of HK2 to eliminate the predicted binding sites (Fig. 3a). This altered luciferase reporter was unaffected by the overexpression of miR-125a (Fig. 3b).

We then evaluated the correlation between miR-125a and HK2. We assessed the protein level of HK2 in HepG2 and Huh-7 cells after transfecting with miR-125a mimics and inhibitors in normaxia. As expected, overexpression of miR-125a significantly increased cellular levels of miR-125a (Supplementary Fig. 2a and b) and reduced HK2 protein levels in HCC cells (Figs 3c–f and 2c), whereas knockdown of miR-125a dramatically reduced cellular levels of miR-125a (Supplementary Fig. 2a and b) and increased the level of HK2 protein in HCC cells (Fig. 3c–f). Taken together, these data indicate that miR-125a can directly target HK2.

### miR-125a is inversely correlated with the protein level of HK2 in tumor tissues of patients with HCC

**Figure 4:**
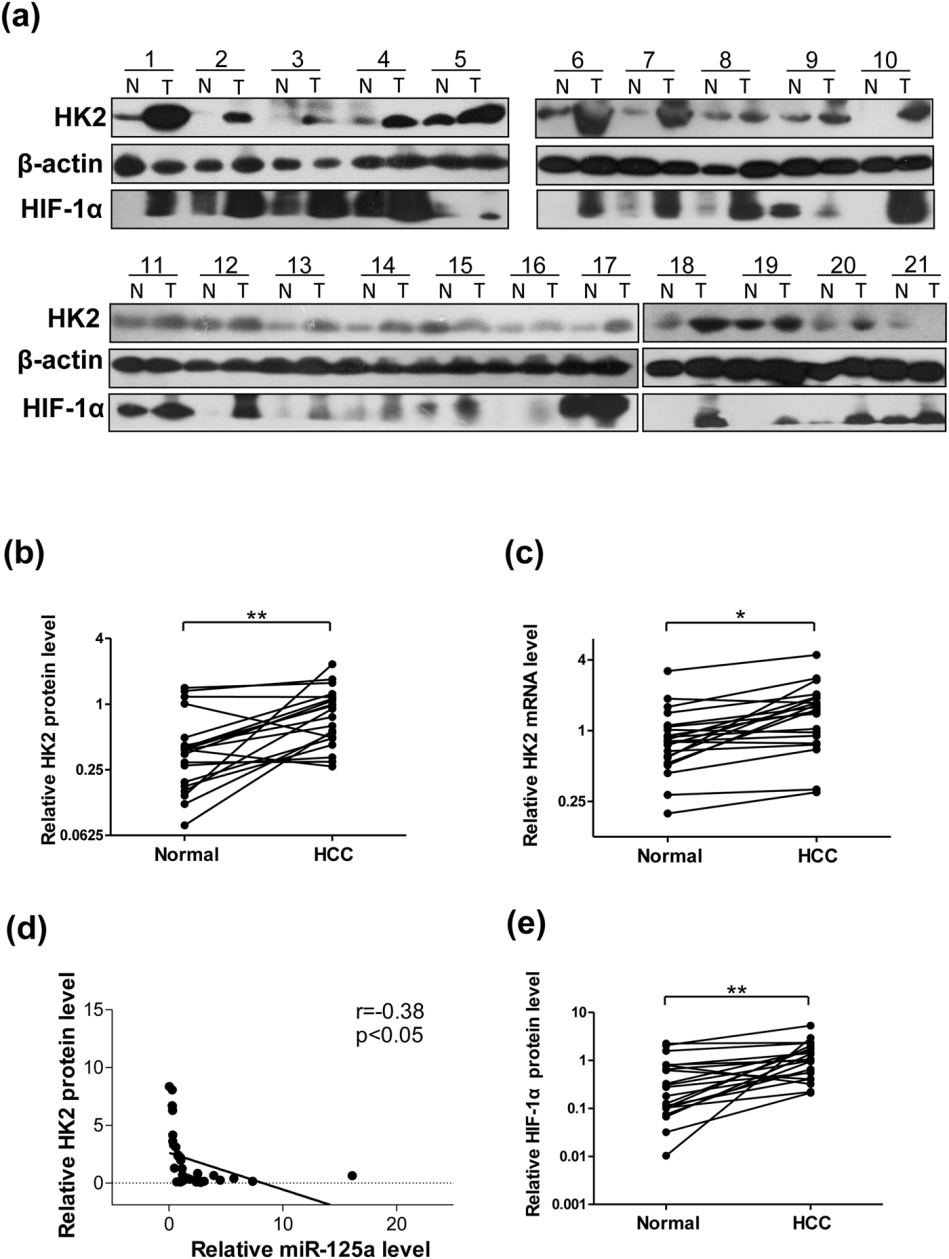
The expression of miR-125a and HK2 is correlated in human HCC specimens. (**a**) Protein level of HK2 and HIF-1α was determined using Western blotting in 21 pairs of human HCC samples. β-actin was used as an internal control. (**b**) Quantitative analyses of the protein level of HK2 in 21 pairs of HCC samples. (**c**) Quantitative analyses of the mRNA level of HK2 in 21 pairs of HCC samples. (**d**) Pearson’s correlation scatter plot of the fold changes of miR-125a and HK2 protein in paired HCC tissues. (**e**) Quantitative analyses of the protein level of HIF-1α in 21 pairs of HCC samples.

To test the clinical relevance, tumor tissues and background liver tissues obtained from 21 paired patients with HCC (Table 1) were analyzed. Representative histological section figures of HCC samples and adjacent noncancerous tissue were shown in Supplementary Fig. 3. By measuring the levels of HIF-1α protein in the 21 pairs of HCC samples and adjacent noncancerous tissues, we found that HIF-1α were upregulated in the tumor tissues (Fig. 4a and e), indicating that HCC tissues were anoxic. Similarly, protein levels of HK2 were upregulated in the cancer tissues (Fig. 4a and b), but mRNA levels of HK2 did not differ as much as the protein levels between the cancerous and noncancerous tissues (Fig. 4c). Finally, the correlation was analyzed between the expression levels of miR-125a and HK2 protein in primary HCC specimens. As shown in Fig. 4d, miR-125a is inversely correlated to HK2. The value of negative correlation coefficient is −0.38.

**Table 1:** Clinical features of the studied patients with hepatocellular carcinoma.

Number	Age	Gender	Pathological Stage	HBV infection
Case #1	57	male	III	HBV+ (93.57)
Case #2	61	male	II	HBV+ (>225)
Case #3	75	male	I	−(0.001)
Case #4	40	male	III	HBV+ (>250)
Case #5	50	male	I~II	HBV+ (31.83)
Case #6	48	male	I	HBV+ (>225)
Case #7	42	male	II~III	HBV+ (9.102)
Case #8	53	male	III	HBV+ (11.687)
Case #9	68	male	II	−(0.001)
Case #10	45	male	III	HBV+ (>250)
Case #11	54	male	III	HBV+ (>250)
Case #12	66	male	II~III	HBV+ (9.102)
Case #13	46	male	II	−(0.001)
Case #14	61	male	I	HBV+ (133.69)
Case #15	53	male	III	HBV+ (11.687)
Case #16	49	male	II	−(0.001)
Case #17	55	male	I	−(0.001)
Case #18	74	male	I	−(0.001)
Case #19	63	male	II	HBV+ (72.519)
Case #20	60	female	I	HBV+ (250)
Case #21	66	male	III	HBV+ (152.283)

HBV, hepatitis B virus. Pathological Stage was classified according to Edmondson-Steiner^48^.

### The miR-125a/HK2 axis is functionally important for regulating HCC cell glycolysis, growth and apoptosis

**Figure 5:**
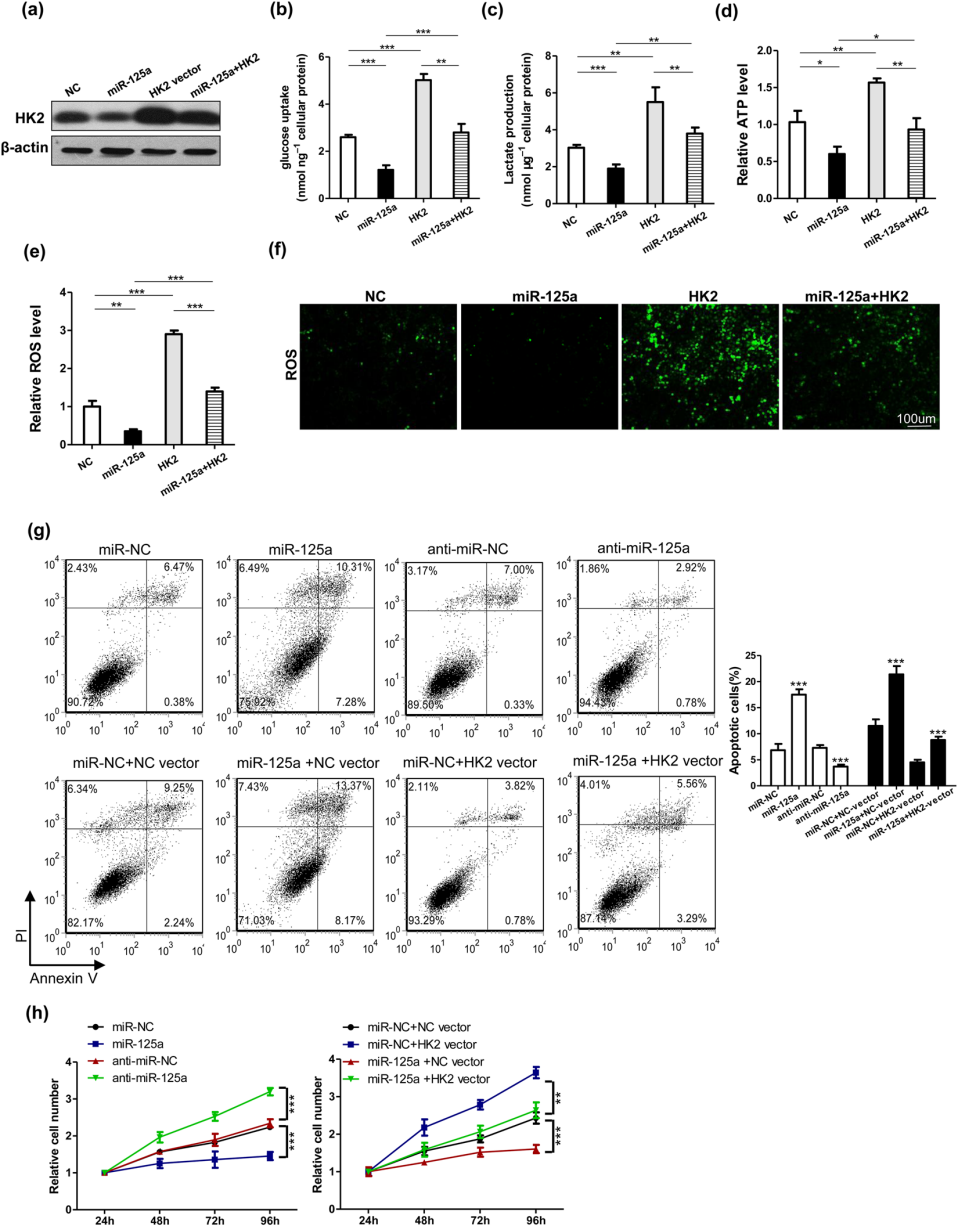
The miR-125a/HK2 axis is functionally important for regulating HCC cell glucose metabolism, growth and apoptosis. HepG2 were transfected with miR-NC, miR-125a mimics, HK2 vector and miR-125a mimics plus HK2-vector in normaxia. (**a**) Representative Western blotting analyses of HK2. The uptake of glucose (**b**), the production of lactate (**c**), cellular levels of ATP (**d**) and ROS (**e**) were evaluated. (**f**) ROS was observed by fluorescence micrography. (**g**) Apoptosis of HepG2 cells under various transfections was analyzed using flow cytometry. (**h**) Viabilities of HepG2 cells under various transfections were determined using a CCK-8 assay at various time points.

To further examine the regulatory role of the miR-125a/HK2 axis in glycolysis, we transfected HepG2 cells and found that co-transfected HK2-expressing plasmids counteract the inhibitory effect of miR-125a mimics on HK2 (Fig. 5a). Moreover, the uptake of cellular glucose, the production of lactate, the cellular levels of ATP and ROS in miR-125a-overexpressing group were much lower (Fig. 5b–f). Furthermore, co-transfecting HK2-expressing plasmids rescued the down-regulatory effect of miR-125a on these metabolic parameters (Fig. 5b–f).

As the Warburg effect is a metabolic hallmark of cancer, tumor cells modify their metabolic pathway from oxidative phosphorylation to aerobic glycolysis to efficiently grow and proliferate^6, 7, 29^. We next assessed cell proliferation and apoptosis in HepG2 cells after transfecting with miR-125a mimics and inhibitors with or without HK2-expressing plasmids. As expected, overexpression of miR-125a increased apoptosis and decreased cell viability in HCC cells (Fig. 5g and h), whereas suppression of miR-125a or overexpression of HK2 increased cell viability and decreased apoptosis (Figs 5g and h and 3). Moreover, co-transfecting miR-125a mimics with the HK2-vector abolished the up-regulation of cell viability and the down-regulation of apoptosis of HepG2 cells (Fig. 5g and h). Conversely, Knockdown of HK2 (Supplementary Fig. 4a–c) promoted cell apoptosis (Supplementary Fig. 4d and e) and decreased cell viability in HCC cells (Supplementary Fig. 4f). Collectively, these results indicate that the miR-125a/HK2 axis is important in regulating glucose metabolism, growth and apoptosis in HCC cells.

### The miR-125a/HK2 axis plays a functional role in regulating the Warburg effect *in vivo*

**Figure 6:**
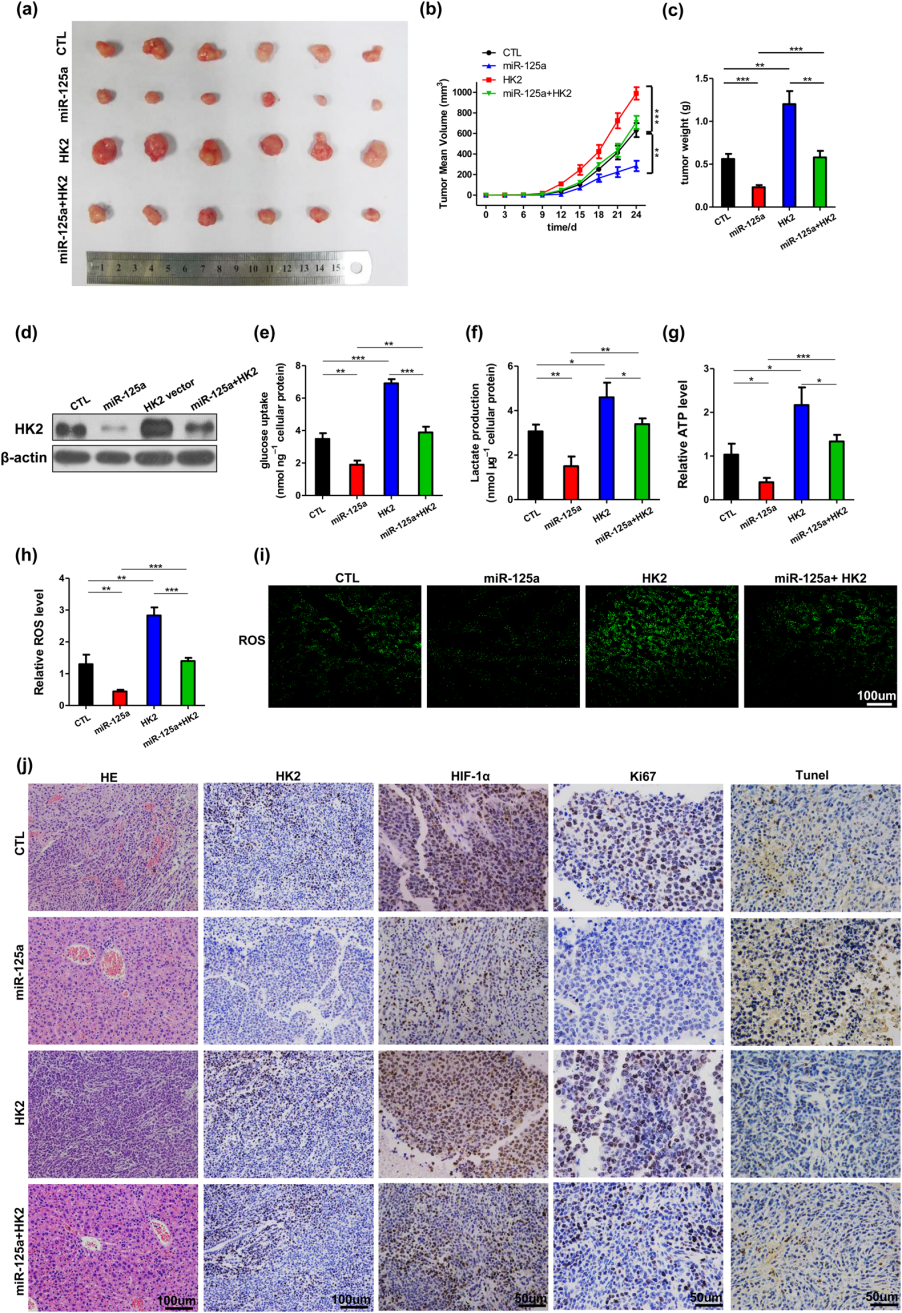
The miR-125a/HK2 axis is of functional importance in regulating the Warburg effect *in vivo*. Mice were divided into four groups according to the implanted HepG2 cells: control cells (CTL), miR-125a-overexpressing cells (miR-125a), HK2-overexpressing cells (HK2), and HK2 plus Lenti-miR-125a cells (HK2 + miR-125a). (**a**) Representative images of tumors. (**b**) The time course of tumor volume. (**c**) The quantitative analysis of tumor weight. (**d**) Western blotting analyses of HK2 proteins in tumors from four groups of mice. The metabolic parameters of the tumors from four groups was measured, the uptake of glucose (**e**), the production of lactate (**f**), cellular levels of ATP (**g**), and ROS (**h**,**i**). (**j**) HE staining, HK2 and HIF-1α immunohistochemical staining, Ki67 and Tunel staining of tumor tissues in four groups of mice. All data are shown as the means ± S.E. obtained from three separate experiments.

We next evaluated the effects of the miR-125a/HK2 axis on the growth of HCC cell xenografts in mice. HepG2 cells were infected with a lentiviral expression vector to express miR-125a. Efficient expression of miR-125a and inhibition of HK2 in HepG2 cells by transfection with lentiviral vector is shown in Supplementary Fig. 5a–c. HepG2 cells were also transfected with HK2 plasmid or miR-125a overexpression lentivirus plus HK2 overexpression plasmid. Then, the cells were implanted subcutaneously into 6-week-old nude mice, and tumor growth was evaluated on day 24 after cell implantation. As shown in Fig. 6a–c, a significant increase in the sizes and weights of the tumors was observed in the HK2-overexpressing group compared to the control group, whereas the overexpression of miR-125a can significantly decrease tumor volume, suggesting that HK2 overexpression can attenuate the suppressive effect of miR-125a on tumor growth. Subsequently, total RNA and protein were extracted from each xenograft and used to evaluate the expression levels of miR-125a and HK2. After 24 days of xenograft growth *in vivo*, tumors from the mice in the miR-125a overexpression group showed a significant increase in the expression of miR-125a (Supplementary Fig. 5d), and displayed reduced HK2 protein levels compared with the control group (Figs 6d and 5e). Tumors with both miR-125a and HK2 overexpression exhibited significantly higher levels of HK2 compared to tumors with miR-125a overexpression (Figs 6d and 5e). Consistent with this, immunohistochemical studies also revealed the presence of lower levels of HK2 and HIF-1α in the group implanted with miR-125a -overexpressing cells (Fig. 6j).

Next, we investigated whether the miR-125a/HK2 axis inhibits HCC xenograft growth through regulating tumor glycolysis. Tumors from 4 groups were finely minced, grinded, and digested, filtered and finally obtained tumor cells. We measured the metabolic parameters of the tumors from four groups. The uptake of glucose, the production of lactate, the levels of ATP and ROS of miR-125a-overexpressing group were much lower, and those of HK2-overexpressing group were much higher, than those of the control group (Fig. 6e–i). Moreover, co-transfection of these vectors significantly rescued the impact of miR-125a on glycolysis in tumors (Fig. 6e–i). These results together indicate that miR-125a suppresses HCC tumor glycolysis by inhibiting HK2. In addition, hematoxylin and eosin (H&E) staining of the xenograft tissues also showed less cell mitosis in tumors of the miR-125a-overexpressing group than in tumors of the control group (Fig. 6j). Xenografts with both miR-125a and HK2 overexpression exhibited reduced cell mitosis and increased cell apoptosis compared to xenografts with HK2 overexpression (Fig. 6j), suggesting that miR-125a overexpression could attenuate the tumor-promoting effect of HK2. Finally, Ki67 and Tunel staining of the tumor sections showed that the miR-125a group contained fewer Ki67-positive and more Tunel-positive cells than those of the control, HK2, and miR-125a plus HK2 groups and the HK2 group had more Ki67-positive and less Tunel-positive cells than the control and miR-125a plus HK2 groups (Figs 6j and 5f and g). These results are consistent with the findings made in the *in vitro* assays and firmly validate the biological role of the miR-125a/HK2 axis in HCC progression.

## Discussion

Energy metabolism reprogramming, such as with elevated glycolysis, is a common feature of cancer. Tumor cells rapidly adjust their energy source from oxidative phosphorylation to glycolysis in order to efficiently proliferate in a hypoxic environment^5^. miRNAs have been reported to play important roles in regulating tumor cell growth, cell cycle process, migration, angiogenesis and metastasis, depending on the function of their target genes^30, 31^, but the role of miRNAs in hypoxia-altered cancer cell metabolism remains incompletely understood, especially in HCC. Recent studies indicate that breast cancer cell-secreted miR-122 suppresses glucose uptake by niche cells and facilitates metastasis by targeting pyruvate kinase^32^, and miR-155/miR-143 cascade controls glycolysis by regulating HK2 expression in breast cancer cells^14^, demonstrating that miRNA is of functional importance in regulating glucose metabolism in cancer cells.

Previous studies have shown that miR-125a plays a tumor-suppressive role in various cancers through negatively regulating tumor oncogenes^33–36^. For example, miR-125a was found to be an independent prognostic factor and inhibit proliferation of gastric cancer^37^ and miR-125a function as tumor suppressor by regulating abnormal activity of SIRT7 in human HCC tumorigenesis^38^. In this study, we observed that hypoxia potently induces the miR-125a down-regulation in HCC cells and tumors. Our data here show that miR-125a robustly suppresses glycolysis in HCC cells. To the best of our knowledge, this is the first report showing that this tumor-suppressive miRNA, miR-125a, also plays a critical role in regulating the Warburg effect in HCC. We identified hexokinase 2 (HK2), which is both highly elevated in rapidly growing cancers and catalyzes the irreversible first step of glucose metabolism and is a key glycolytic enzyme for the Warburg effect^9, 18, 22, 23, 36^, and thus it is reported as a pivotal player in the Warburg effect and is proposed as a metabolic target for cancer therapeutic development^9, 10, 36^, as a novel target of miR-125a and further demonstrated that miR-125a represses glycolysis by targeting HK2 in HCC cells.

Aberrant tumor metabolism supports tumor cell energy requirements and their enormous biosynthetic needs, further promoting cancer progression and metastasis^6, 29, 39^. In this study, we showed that HK2 promotes the growth and inhibits the apoptosis of HCC cells, suggesting that HK2 is oncogenic in HCC. This is in agreement with a recent report showing that HK2 promotes the growth and migration of breast cancer cells^14^ and glioblastoma multiforme^40^. Previous studies suggest that miR-125a exerts its tumor-suppressive function through targeting oncogenes^34^. Our findings here indicated that targeting HK2 to regulate the hypoxia-regulated cancer glucose metabolism switch also contributes to the antitumor activity of miR-125a. These results indicate that the miR-125a/HK2 axis, in addition to regulating glucose metabolism, plays an important role in controlling tumorigenesis in HCC cells. These findings not only further support the notion that cancer cells use the Warburg effect to gain glycolytic intermediates for sustaining fast-proliferation of cancer cells but also add a novel molecular link between tumor biology and tumor metabolism.

In summary, this study not only uncovered the critical roles of hypoxia-down-regulated miR-125a in the Warburg effect of HCC cancer but also explored the molecular mechanisms through which miR-125a regulated HCC glycolysis and progression and identified HK2 as a direct target gene. Regulation of HK2 by miR-125a may explain why the down-regulation of miR-125a during HCC carcinogenesis promotes tumor growth and Warburg effect. This study may provide insight into the molecular mechanism of the glucose metabolism switch during HCC carcinogenesis and open a new avenue for HCC treatment.

## Materials and Methods

### Ethics Statement

All methods and experimental protocols were approved by Nanjing University and were carried out in accordance with the approved guidelines. Written consent was provided by all of the patients, and the Ethics Committee of Jiangsu Cancer Hospital and Research Institute approved all aspects of this study. Informed consent was obtained from all subjects enrolled in the studies that provided the samples. A total of 21 paired HCC cancer and normal adjacent tissues were derived from patients undergoing a surgical procedure at the Jiangsu Cancer Hospital (Nanjing, China). Both tumors and noncancerous tissues were confirmed histologically. The pathological type of each cancer was determined to be infiltrating ductal carcinoma. Tissue fragments were immediately frozen in liquid nitrogen at the time of surgery and stored at −80 °C. The clinical features of the patients are listed in Table 1. The 6-week-old male SCID (severe combined immune deficiency) mice (nu/nu) used in this study were obtained from the Model Animal Research Center of Nanjing University (Nanjing, China) and maintained under specific pathogen-free conditions at Nanjing University and followed the National Institutes of Health guide for the care and use of mice.

### Cell culture

Huh-7, HepG2, 293T cells were obtained from Shanghai Institute of Cell Biology, Chinese Academy of Sciences (Shanghai, China) and maintained in DMEM (Gibco, CA) supplemented with 10% fetal bovine serum (FBS) and 1% penicillin–streptomycin within a humidified atmosphere containing 5% CO2 at 37 °C. For hypoxia stimulation, cells were transferred to a hypoxia chamber with 1% oxygen. Cells using for functional and mechanism studies in this study were tested and authenticated using short tandem repeat (STR) method by Shanghai Institute of Cell Biology.

### miRNA-Related Reagents, Small Interfering RNAs, and Transfection

An amount of 100 pmol miR-125a mimics or inhibitors (sequence: TCCGGTTGGGTCTCGGG) were transfected into Huh-7 or HepG2 cells in 6-well plates with Lipofectamine 2000 (Invitrogen) according to the manufacturer’s instruction. HK2 small interfering RNA (sequence: GGAGGAUGAAGGUAGAAAUUU) were purchased from Ruibo Company (Guangzhou,China). For the HK2 overexpression assay, a pcDNA3.1 vector was designed to specifically express the open reading frame (ORF) of human HK2 containing full-length 3′-UTR and purchased from GenScript (Nanjing, China).

### RNA isolation and real-time quantitative PCR

Total RNA extraction, Reverse transcription and TaqMan real-time polymerase chain reaction (PCR) for miRNAs were performed as described previously^41^. Real-time PCR for mRNA detection were performed using SYBR Green PCR Master Mix (Ambion). The sequences of the primers used were as follows: HK2 mRNA (sense): 5′-AAGGCTTCAAGGCATCTG-3′, HK2 mRNA(antisense): 5′-CCACAGGTCATCATAGTTCC-3′, β-actin(sense): 5′-GGCACCCAGCACAATGAAG-3′ and β-actin(antisense): 5′-GCCGATCCACACGGAGTA-3′. For the analysis of glucose metabolism gene expression, cDNA was synthesized using RT^2^ First Strand Kit (Qiagen). The expression of 84 genes involved in glucose metabolism were then analyzed using RT^2^ Profiler PCR Human Glucose Metabolism Array (PAHS-006Z, Qiagen) as described previously^42^. For data analysis, the RT^2^ Profiler PCR Array software package was used and statistical analyses were performed online at https://www.qiagen.com/cn/shop/genes-and-pathways/technology-portals/browse-qpcr/qrt-pcr-for-mrna-expression/data-analysis/geneglobe-data-analysis-center/?akamai-feo=off.

### Antibody and Western Blotting

Cellular protein were extracted as described previously^41^. Antibodies against HK2 (Cell Signaling Technology) were used for blotting. β-actin (Cell Signaling Technology, CA) served as an internal control.

### *In situ* hybridization

*In situ* hybridization of human HCC tissues using an miRCURY LNA™ microRNA probe directed against has-miR-125a-5p (probe Sequence: CACAGGTTAAAGGGTCTCAGGGA) synthesized by Exiqon was performed as described previously^43, 44^.

### Luciferase reporter assay

For the luciferase reporter assay, p-MIR-REPORT plasmids were designed to contain the 3′-untranslated region (UTR) of human HK2 and purchased from GenScript (Nanjing, China). For the mutant assay, the binding motif CUCAGGGA was replaced with GAGTCCCT. 293T cells were cultured in 24-well plates, and each well was transfected with 0.1 μg of firefly luciferase reporter plasmid, 0.1 μg of β-galactosidase (β-gal) expressing plasmid (Ambion) and miR-125a mimics (25 pmol) or negative control using Lipofectamine 2000 (Invitrogen) as described previously^45, 46^. The β-gal plasmid was used as a transfection control. Luciferase activity was measured 24 h after transfection using a luciferase assay kit (Promega, Madison, WI, USA).

### Measurement of lactate production and glucose uptake and ATP production

The extracellular lactate was measured using the cell culture medium with lactate assay kit (BioVision, #K607–100). Intracellular glucose was measured using cell lysates with glucose assay kit (BioVision, #K606–100). ATP levels were measured using an ATP assay kit (Celltiter-Glo Luminescent Cell Viability Assay, Promega). The uptake of glucose, the production of lactate and the levels of ATP were all measured according to the manufacturer’s instruction as described previously^15, 47^. For detecting the uptake of glucose and the production of lactate, the culture supernatants of tumor cells with different treatments were collected and the fresh culture media was used as control. Equal amounts (2–10 µl) of tested samples were added to a 96-well plate and the volume were then adjusted to 50 µl/well with Glucose or Lactate Assay Buffer respectively. Meantime, the standard curve was prepared with the same protocol. The reaction were incubated in dark for 30 min at 37 °C. Finally, the absorbance (OD 570 nm) or Fluorescence (Ex/Em = 535/590 nm) were measured by microplate reader. The uptake of Glucose was determined by subtracting the concentration of glucose in tested samples from the initial concentration of glucose in fresh culture media. The production of lactate was determined referring standard curve. Considering the cell number of every sample may be different, all the concentration of glucose or production of lactate were finally normalized to the protein concentration.

### Measurement of ROS production

HepG2 cells and tumor slices were harvested, washed with serum-free culture medium and incubated with 5umol/l dichlorofluorescein diacetate (DCFH-DA, Beyotime) at 37 °C for 30 min in the dark. Then the cells and tumor slices were harvested, washed and resuspended in serum-free culture medium. DCF fluorescence distribution was recorded. (DCF: excitation wavelength 488 nm and emission wavelength 525 nm).

### Immunohistochemistry staining

Immunohistochemistry staining was done as described previously^18^. To evaluate tumor histological changes, tumor sections were processed for H&E staining as described previously^45^. Immunohistochemical staining of the paraffin sections was performed using a microwave-based antigen retrieval technique, specimen slides were incubated overnight at 4 °C with primary antibodies that were raised against Ki-67 (Cell Signaling Technology, USA) and HK2 (Cell Signaling Technology).

### Animal model

Untreated HepG2 cells or HepG2 cells infected with miR-125a overexpressing lentiviral vector or transfected with the HK2 expressing plasmid were injected subcutaneously into SCID mice (1 × 10^6^ cells per mouse, 6 mice per group). Tumor volume was measured every 3 days for 24 days after inoculation. The length, width and height of the tumors were measured with digital calipers and the ellipsoid volume was calculated using the following formula: Volume = π/6 × (length) × (width) × (height). The tumor tissues were fixed in 10% formalin for 24 h for further H&E and immunohistochemical staining. For measuring the metabolic state of tumor, tumors from 4 groups were finely minced, grinded, and digested with RPMI-1640 containing 1 mg/ml collagenase D and 100 U/ml DNaseI for 45 minutes at 37 °C, then passed through 70-mm nylon cell strainer, centrifuged to get rid of large pieces of tissues, and depleted the red blood cells. Finally, tumor-cell suspensions were obtained and cultured for determining the metabolic parameters.

### Statistical analysis

Each experiment is representative of at least three independent experiments. The data are presented as the means ± SEM of at least three independent experiments. Differences between groups were analyzed using Student’s t-test. Differences between more than two groups were analyzed using ANOVA. Throughout the text, figures, and figure legends, the following terminology is used to denote statistical significance: *P < 0.05; **P < 0.01; and ***P < 0.001.

## Electronic supplementary material

The miR-125a/HK2 axis regulates cancer cell energy metabolism reprogramming in hepatocellular carcinoma
