# Supplementary material for: The miR-125a/HK2 axis regulates cancer cell energy metabolism reprogramming in hepatocellular carcinoma
Source: Sci Rep. 2017 Jun 8;7:3089. doi: 10.1038/s41598-017-03407-3 (PMC5465066; doi:10.1038/s41598-017-03407-3)
Supplement: Supplementary file 1 — The miR-125a/HK2 axis regulates cancer cell energy metabolism reprogramming in hepatocellular carcinoma [file 41598_2017_3407_MOESM1_ESM.doc]

**Supplementary Data to:**

**The miR-125a/HK2 axis regulates cancer cell energy metabolism** **reprogramming in**

**hepatocellular carcinoma**

Fangfang Jin^1*^, Yanbo Wang^1*^, Yanan Zhu^1*^, Shan Li^1^, Ying Liu^1^, Cheng Chen^3^, Xiaohua Wang^2#^, Ke Zen^1#^, and Limin Li^1#^

^1^State Key Laboratory of Pharmaceutical Biotechnology, Nanjing University Advanced Institute of Life Sciences, Jiangsu Engineering Research Center for MicroRNA Biology and Biotechnology, Nanjing University, Nanjing, Jiangsu 210093. ^2^ Department of Chemotherapy, Jiangsu Cancer Hospital and Research Institute, Nanjing, Jiangsu 210009. ^3^Department of Radiotherapy, Nanjing Medical University Affiliated Cancer Hospital, Cancer Institute of Jiangsu Province, Nanjing, Jiangsu 210009.

**Table of content**

[Supplementary Figure 1. Hypoxia induced HIF-1α expression in HepG2 and Huh-7 cells. 2](#__RefHeading___Toc456197085)

[Supplementary Figure 2. Transfection of miR-125a mimics or inhibitors can effectively upregulate or downregulate the expression of miR-125a in Huh-7 and HepG2 cells. 2](#__RefHeading___Toc456197087)

[Supplementary Figure 3. Representative histological section figures of HCC samples and adjacent noncancerous tissue. 3](#__RefHeading___Toc456197087)

[Supplementary Figure 4 Overexpression of HK2 decreased apoptosis and increased cell viability in HepG2 cells... 4](#__RefHeading___Toc456197088)

[Supplementary Figure 5. Stably expressing miR-125a inhibits HK2 in vitro and in vivo. 5](#__RefHeading___Toc456197088)

[Supplementary Figure 6. Full gel images of Western blots. 6](#__RefHeading___Toc456197088)

**
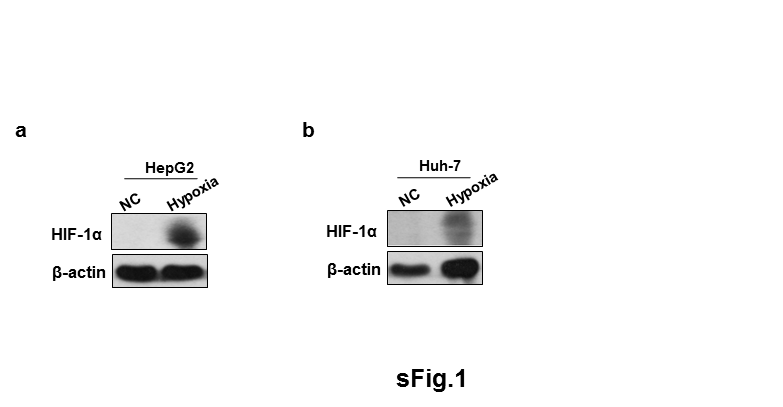
**

**Supplementary Figure 1. Hypoxia induced HIF-1α expression in HepG2 and Huh-7 cells.** HepG2 cells (a) and Huh-7 cells (b) were treated with hypoxia for 48 h. HIF-1α levels were measured by Western Blotting..

**
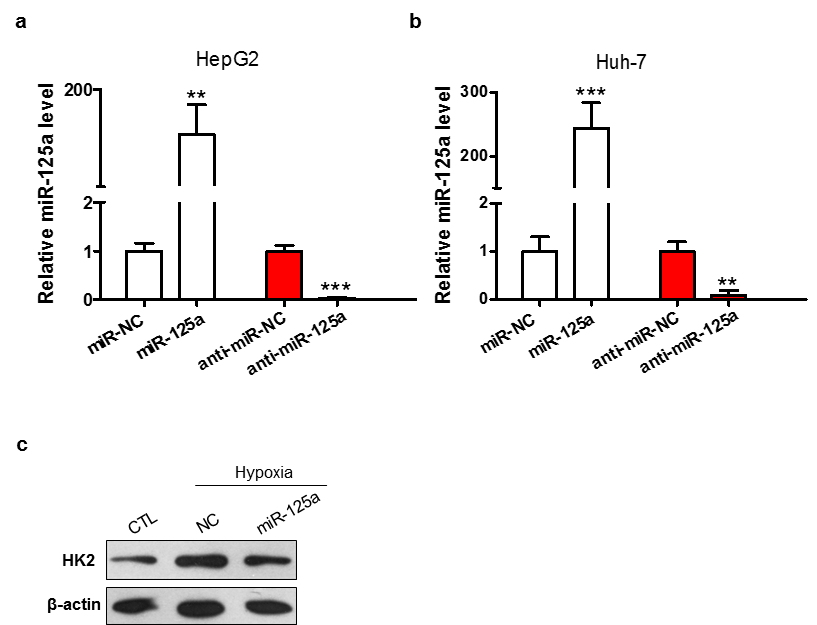
**

**Supplementary Figure 2. Levels of miR-125a in HepG2 and Huh-7 cells transfected with miR-125a mimics and inhibitors.** HepG2 cells (a) and Huh-7 cells (b) were transfected with miR-125a mimics and inhibitors. miR-125a levels were detected 24 h later respectively. (c) HepG2 cells were transfected with NC, miR-125a mimics under nomoxia/hypoxia, HK2 level was measured by Western Blotting. ** P < 0.01, *** P < 0.001.


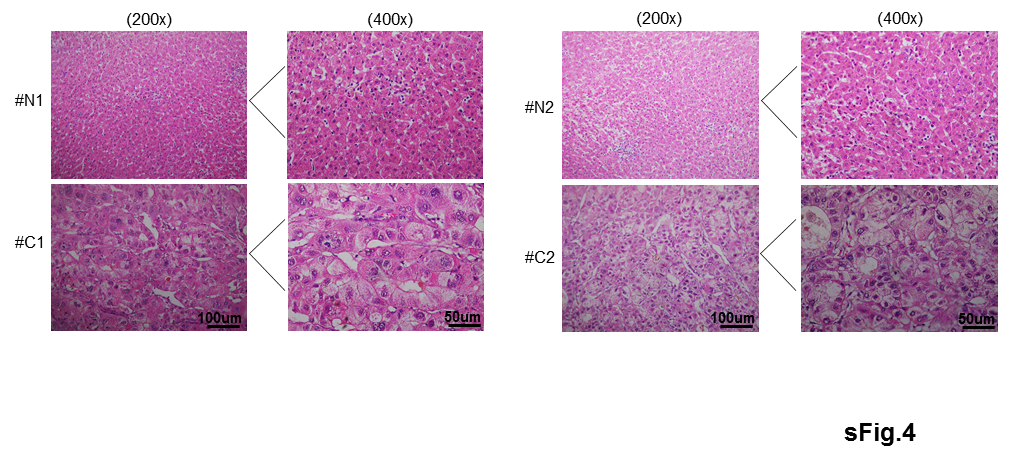


**Supplementary Figure 3.** Representative histological section figures of HCC samples and adjacent noncancerous tissue.


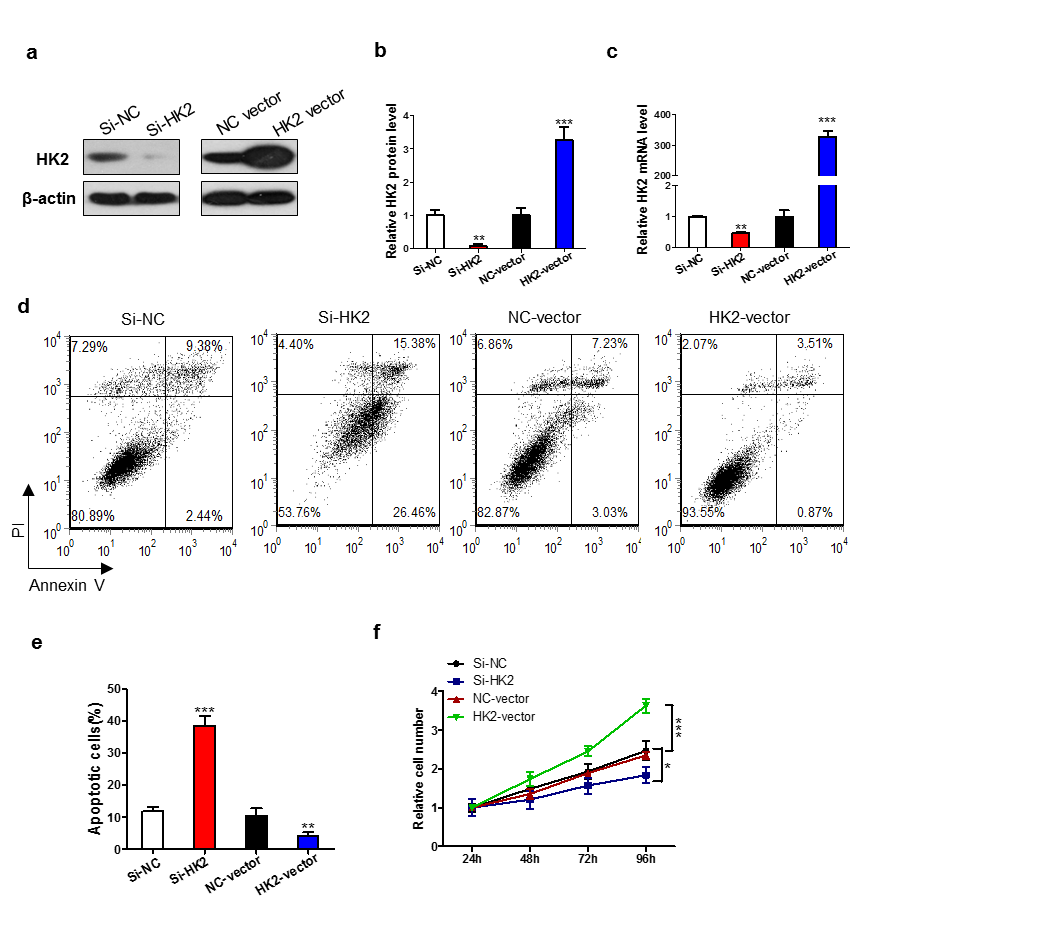


**Supplementary Figure 4. Overexpression of HK2 decreased apoptosis and increased cell viability in HepG2 cells.** HepG2 cells were transfected with random siRNA oligonucleotides (Si-NC) or siRNA of HK2 (Si-HK2), empty vector (NC vector), HK2 expressing plasmids (HK2 vector). **(a)** HK2 protein level was determined using Western blotting with different transfection. β-actin was used as an internal control. **(b)** Quantitative analyses of the protein level of HK2. **(c)**The level of HK2 mRNA was detected by RT-qPCR in HepG2 cells with different transfection. Data are shown as mean ±S.E from three separate experiments. **(d)** Cell apoptosis of HepG2 cells under different transfection was analyzed using flow cytometry. **(e)** Quantitative analyses of cell apoptosis. **(f)** The cell viability of HepG2 cells under different transfections were determined using a CCK-8 assay. *P<0.05;** P < 0.01; *** P < 0.001.


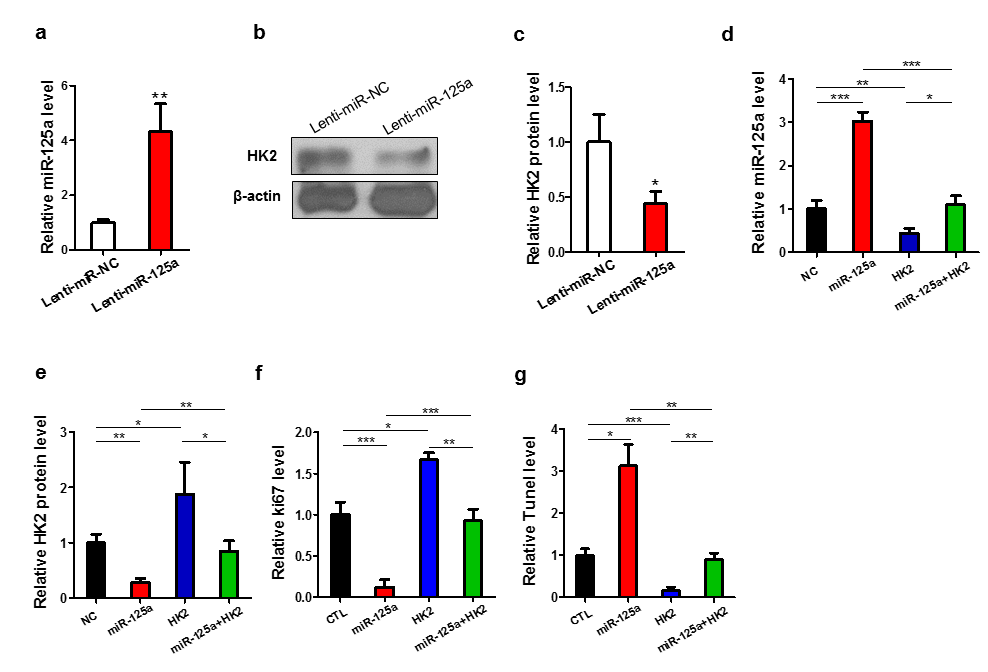


**Supplementary Figure 5. Stably expressing miR-125a inhibits HK2 in vitro and in vivo.** HepG2 cells stably expressing miR-125a were constructed using lentivirus-packaged miR-125a (Lenti-miR-125a), and lentivirus-packaged empty vector (Lenti-miR-NC) was used as the control. **(a)** Relative miR-125a level in HepG2 cells expressing Lenti-miR-NC or Lenti-miR-125a. **(b)** Protein levels of HK2 in HepG2 cells expressing Lenti-miR-NC or Lenti-miR-125a. **(c)**The histogram represents a quantitative analysis of HK2 protein levels. **(d)** Quantitative analysis of miR-125a levels in tumors of 4 mouse groups. **(e)**Western blotting analyses of HK2 proteins in the tumors of mice. **(f)** Quantitative analysis of Ki67 staining of the tumor sections from 4 groups. **(g)** Quantitative analysis of Tunel staining of the tumor sections in 4 groups. Statistical data are presented as the means ±S.E. from three independent experiments. *P<0.05;** P < 0.01; *** P < 0.001.


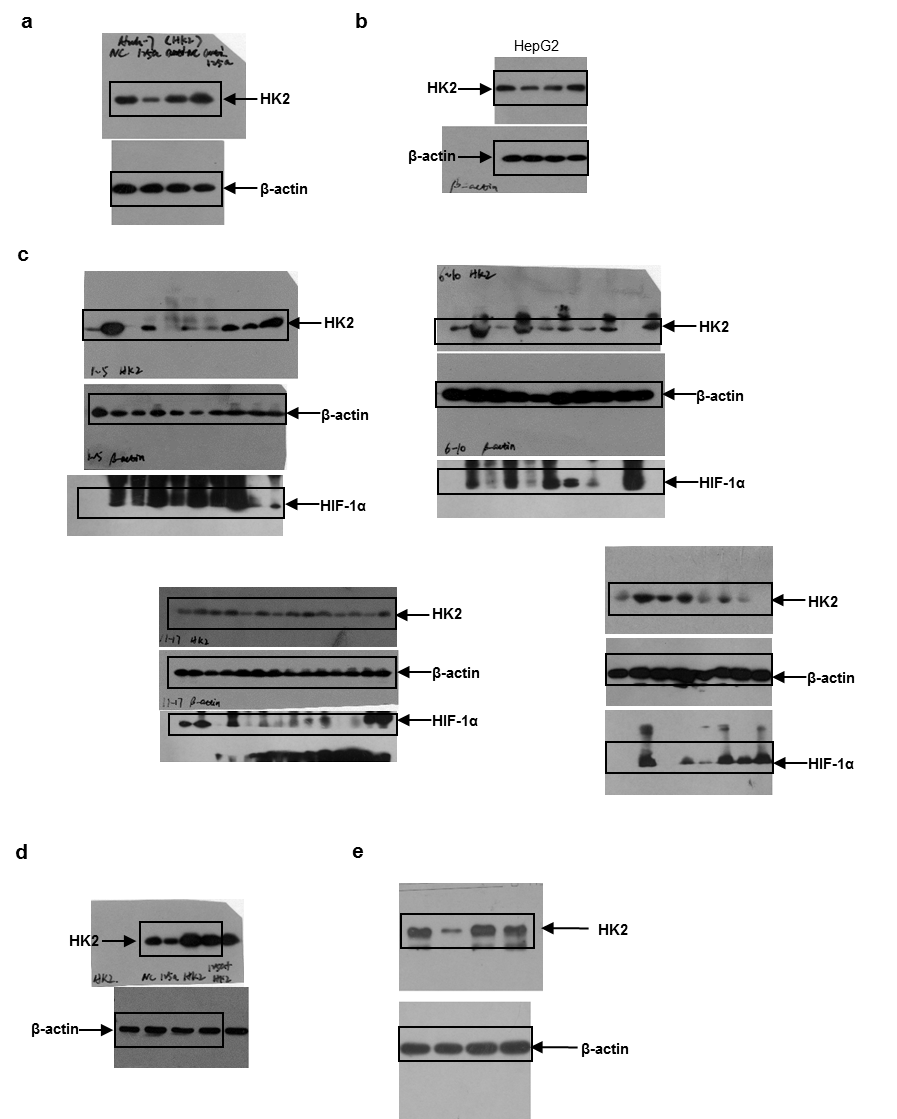


**Supplementary Figure 6. Full gel images of Western blots. (a)**The full-size images corresponding to those shown in Figure 3c, **(b)** Figure 3e, **(c)** Figure 4a, **(d)** Figure 5a, **(e)** Figure 6d.
